# Microglial Hyperreactivity Evolved to Immunosuppression in the Hippocampus of a Mouse Model of Accelerated Aging and Alzheimer’s Disease Traits

**DOI:** 10.3389/fnagi.2020.622360

**Published:** 2021-01-28

**Authors:** Patricia Molina-Martínez, Rubén Corpas, Elisa García-Lara, Marta Cosín-Tomás, Rosa Cristòfol, Perla Kaliman, Carme Solà, José Luis Molinuevo, Raquel Sánchez-Valle, Anna Antonell, Albert Lladó, Coral Sanfeliu

**Affiliations:** ^1^Institut d’Investigacions Biomèdiques de Barcelona (IIBB), Consejo Superior de Investigaciones Científicas (CSIC), Barcelona, Spain; ^2^Institut d’Investigació Biomèdica August Pi i Sunyer (IDIBAPS), Barcelona, Spain; ^3^Faculty of Health Sciences, Universitat Oberta de Catalunya, Barcelona, Spain; ^4^Alzheimer’s Disease and Other Cognitive Disorders Unit, Department of Neurology, Hospital Clínic, Barcelona, Spain; ^5^Fundació Clínic per a la Recerca Biomèdica, Universitat de Barcelona, Barcelona, Spain; ^6^Centro de Investigación Biomédica en Red de Fragilidad y Envejecimiento Saludable (CIBERFES), Madrid, Spain

**Keywords:** neuroinflammation, SAMP8 mice, autosomal dominant Alzheimer’s disease (ADAD), sporadic early-onset Alzheimer’s disease (sEOAD), triggering receptor expressed on myeloid cells 2 (TREM2)

## Abstract

Neuroinflammation is a risk factor for Alzheimer’s disease (AD). We sought to study the glial derangement in AD using diverse experimental models and human brain tissue. Besides classical pro-inflammatory cytokines, we analyzed chitinase 3 like 1 (CHI3L1 or YKL40) and triggering receptor expressed on myeloid cells 2 (TREM2) that are increasingly being associated with astrogliosis and microgliosis in AD, respectively. The SAMP8 mouse model of accelerated aging and AD traits showed elevated pro-inflammatory cytokines and activated microglia phenotype. Furthermore, 6-month-old SAMP8 showed an exacerbated inflammatory response to peripheral lipopolysaccharide in the hippocampus and null responsiveness at the advanced age (for this strain) of 12 months. Gene expression of *TREM2* was increased in the hippocampus of transgenic 5XFAD mice and in the cingulate cortex of autosomal dominant AD patients, and to a lesser extent in aged SAMP8 mice and sporadic early-onset AD patients. However, gene expression of *CHI3L1* was increased in mice but not in human AD brain samples. The results support the relevance of microglia activation in the pathways leading to neurodegeneration and suggest diverse neuroinflammatory responses according to the AD process. Therefore, the SAMP8 mouse model with marked alterations in the dynamics of microglia activation and senescence may provide a complementary approach to transgenic mouse models for the study of the neuroinflammatory mechanisms underlying AD risk and progression.

## Introduction

Neuroinflammation and peripheral inflammatory conditions associated with aging or some pathological conditions are known risk factors for triggering sporadic Alzheimer’s disease (AD) (Bolós et al., 2017; Tejera et al., 2019). Furthermore, lower peripheral inflammation is one of the markers associated with preserved memory at middle age (Corpas et al., 2019). Accordingly, experimental studies have shown neuroprotection by diverse anti-inflammatory agents against memory loss and AD-like pathology (Corpas et al., 2017; Ettcheto et al., 2017). Furthermore, some observational studies have shown the potential of non-steroidal anti-inflammatory drugs (NSAIDs) for prevention of AD (Wang et al., 2015a). However, a number of clinical trials of NSAIDs and other anti-inflammatory drugs reported to date have shown either their lack of efficacy against AD or serious side effects (Elmaleh et al., 2019). The recent failure of the NSAID naproxen to reduce the progression of presymptomatic AD (Meyer et al., 2019) has prompted the redesign of clinical studies (Hershey and Lipton, 2019) and efforts to broaden the pathways of study in the search for new inflammatory targets (Sharman et al., 2019). The relevance of neuroinflammation in the development of AD is further confirmed by the fact that inflammation-related proteins, including the astroglia-based YKL40 [also known as chitinase 3 like 1 (CHI3L1)] and the microglia-based triggering receptor expressed on myeloid cells 2 (TREM2), are currently considered to be potential AD biomarkers in CSF or blood (Molinuevo et al., 2018). The corresponding genes *CHI3L1* and *TREM2* are widely expressed in cerebral tissue, including the hippocampus and cortical areas sensitive to AD pathology (Guerreiro et al., 2013; Sanfilippo et al., 2019).

In experimental studies, mouse models of AD exhibit a diverse degree of neuroinflammation that generally correlates with the amyloid burden. For instance, the 5XFAD transgenic AD mouse model develops abundant amyloid plaque deposits surrounded by activated astrocyte and microglia (Oakley et al., 2006) in parallel with increased brain levels of pro-inflammatory cytokines (Griñán-Ferré et al., 2016b). However, the neuroinflammatory mechanism may differ between AD mouse models and humans (Hemonnot et al., 2019), which may have contributed to the failure of neuroinflammation targeted drugs in clinical trials of AD (Kodamullil et al., 2017). The senescence-accelerated mouse prone 8 (SAMP8) strain is a spontaneous mouse model of accelerated aging that has been proposed as a model of late-onset sporadic AD (Cheng et al., 2014; Liu et al., 2020). It exhibits neuroinflammation, memory loss and mild AD-like pathology (Álvarez-López et al., 2014; Griñán-Ferré et al., 2016a; Ito et al., 2020). At the systemic level, SAMP8 shows increased concentrations of circulating pro-inflammatory factors and increased activation of inflammatory pathways in several organs in comparison with the control strain senescence-resistant mouse 1 (SAMR1) (i.e., Miró et al., 2017; Fernández-García et al., 2019). Furthermore, we have previously demonstrated that cell cultures of SAMP8 embryonic or neonatal brain maintain the pathogenic dysfunctions found in the adult mouse brain (García-Matas et al., 2008; Díez-Vives et al., 2009; Cristòfol et al., 2012). Therefore, SAMP8 may be a good model in which to study the mechanisms underlying the risk of sporadic AD associated with age-related inflammation. Moreover, systemic inflammation may induce or aggravate neuroinflammation. In this regard, peripheral administration of lipopolysaccharide (LPS) from bacterial endotoxin is widely used to induce inflammatory responses in mice for modeling neurodegeneration processes (for a review see: Catorce and Gevorkian, 2016). LPS is the major component of the outer membrane leaflet of Gram-negative bacteria. A structural motif of LPS is recognized by the innate immune cells as one of the pathogen-associated molecular patterns (PAMPs) that stimulate Toll-like receptors (TLRs); LPS mainly stimulates TLR4. The LPS/TLR4 signaling pathway may trigger potent immune responses (Lu et al., 2008). In brief, LPS-activated peritoneal macrophages and dendritic cells of the innate immune system produce Interleukin 1 (IL1α and IL1β), a first-line mediator in the signaling cascade that would help to fight the infection; this includes vagal stimulation to induce sickness behavior, and further synthesis of IL1β in cells lying outside the blood–brain barrier that would reach brain target cells (mainly microglia, but also endothelial cells and macrophage-like cells among others) to induce prostaglandins and pro-inflammatory cytokines (Konsman et al., 2002). A comparable response of transient sickness behavior and microglial activation is also elicited in humans after a single peripheral administration of LPS (Schedlowski et al., 2014; Sandiego et al., 2015), thus giving value to the LPS challenge in experimental models. Besides changes in the classical pro-inflammatory cytokines and nitric oxide pathways, analysis of the proposed AD biomarkers CHI3L1 and TREM2 may help us to understand the mechanisms involved in neuroinflammatory changes.

We aimed to analyze the reactive gliosis of SAMP8 mouse brain and those of astrocytes and microglia *in vitro*, either in basal conditions or after an acute injury with LPS. Using SAMR1 and SAMP8 mice in adulthood and advanced age we aimed to discern age-related changes. Next, we analyzed *CHI3L1* and *TREM2* gene expression in several scenarios, including the transgenic mouse 5XFAD and post-mortem human samples with advanced AD pathology. A study of these genes in the hippocampus of SAMP8 and 5XFAD mice and in the posterior cingulate cortex of human patients with sporadic early-onset AD (sEOAD) and autosomal dominant AD (ADAD) may help reveal the neuroinflammatory mechanisms underlying AD risk and progression.

## Materials and Methods

### Animals

The senescence-prone SAMP8 mouse strain and the control senescence-resistant SAMR1 strain were developed by selective breeding at Kyoto University (Takeda et al., 1994). SAMP8 mice show accelerated brain senescence with cognitive impairment, neuroinflammation, oxidative stress, traits of amyloid and tau pathologies, and epigenetic alterations (López-Ramos et al., 2012; Cosín-Tomás et al., 2014; Griñán-Ferré et al., 2018). Male SAMP8 and SAMR1 mice aged 6 and 12 months were used for this study. Six-month-old SAMP8 mice show the full pathological phenotype, whereas 12-month-old mice are near the mean survival age and have been poorly analyzed (Porquet et al., 2013). Heterozygous transgenic AD mice of the strain 5XFAD (Oakley et al., 2006) and their wild type siblings (WT), 9-month-old males, were used for selected analysis. All mice were bred at the Animal House of the University of Barcelona (UB, Barcelona, Spain). First progenitors of the SAMP8/SAMR1 strains and 5XFAD were obtained from Harlan (Envigo, Barcelona, Spain) and from Jackson Laboratory (Bar Harbor, ME, United States), respectively. Animals were maintained under standard laboratory conditions of food and water *ad libitum*, 22 ± 2°C, and 12 h:12 h light-dark cycle. All experimental protocols and procedures were approved by the local Ethics Committee for Animal Experimentation (CEEA, UB; DAAM 7136 and DAAM 9323), in accordance with the Decree 214/97 of the Generalitat de Catalunya, Spanish legislation and the European Union Directive 2010/63/EU for animal experiments.

### Glial Cell Cultures

Astrocytes and microglia cultured from neonatal SAMP8 neocortex show senescent and pathological traits that might greatly contribute to pathological brain aging in these mice (García-Matas et al., 2008, 2015; Díez-Vives et al., 2009). Here we used mixed glial cultures enriched in astrocytes and almost pure microglia cultures to discern inflammation mechanisms at the cellular level.

Mixed glial cultures enriched in astrocytes were prepared from the cerebral cortices of 2-day-old SAMP8 and SAMR1 mice as previously described (Sola et al., 2011). Briefly, brains were dissected free of the meninges, diced into small cubes and dissociated by incubation with a 0.5% trypsin-EDTA solution (Gibco) for 25 min. Cells were seeded at 5 × 10^4^ cells/cm^2^ in multi-well plates or on glass coverslips in DMEM supplemented with 2.5 mM glutamine, 100 μg/mL gentamycin and 20% fetal bovine serum (FBS) at 37°C in a humidified incubator with 5% CO_2_. The culture medium was changed every 3–4 days and FBS was progressively lowered to 10% during the first 2 weeks of culture. Experiments were routinely carried out at 21 days *in vitro*. Established mixed glia cultures of both SAMR1 and SAMP8 consisted of 85–90% astrocytes, 10–15% microglia, and 0.1–1% oligodendroglia.

Microglial cultures were obtained from mature mixed glial cultures prepared as described above, followed by mild trypsinization to detach an upper layer of mixed glia while maintaining a bottom layer of microglia attached to the plate (Saura et al., 2003). Cultures were used immediately. Established microglia cultures of both SAMR1 and SAMP8 consisted of >98% microglial cells.

### Lipopolysaccharide Treatment

*In vivo*, SAMP8 and SAMR1 mice were subjected to a pro-inflammatory injury by administration of LPS from *Escherichia coli* (serotype 026:B6; Sigma, Saint Louis, MO, United States). LPS was dissolved in sterile physiological saline solution to obtain 100 μg in 0.1 mL for intraperitoneal injection. Mice received a dose of 3 mg per kg body weight or the equivalent volume of vehicle. The experiment was terminated after 3 h of treatment with LPS or vehicle. The experimental groups were as follows: SAMR1-Control 6 months (*n* = 10); SAMR1-Control 12 months (*n* = 7); SAMP8-Control 6 months (*n* = 6); SAMP8-Control 12 months (*n* = 3); SAMR1-LPS 6 months (*n* = 10); SAMR1-LPS 12 months (*n* = 7); SAMP8-LPS 6 months (*n* = 5); and SAMP8-LPS 12 months (*n* = 4). SAMP8 mice have a reduced lifespan with median and maximum life expectancies of 10 and 16 months, respectively (Porquet et al., 2013). Therefore, older SAMP8 mice were visually inspected for overall health appearance prior to their inclusion in the study.

*In vitro*, pro-inflammatory injury was induced in glial cell cultures by adding LPS at the final concentration of 100 ng/mL in the culture medium. Interferon-γ (IFN; Sigma) at the final concentration of 0.1 ng/mL was simultaneously added to potentiate the effects of LPS (Straccia et al., 2011). Experiments were terminated after 24 h of exposure to LPS + IFN or vehicle (saline). Conditioned culture media and/or cells were immediately collected for further assays. All experiments were performed in cells from at least *n* = 3 independent primary cultures.

### Sickness Behavior

Decreased motivation to engage in social exploratory behavior is used to assess sickness behavior induced by an infection (Dantzer, 2001). Sickness behavior shows the adaptive reorganization of an animal’s priorities. SAMP8 and SAMR1 mice were subjected to the social behavior test immediately before administration of LPS or vehicle and again 3 h later. A juvenile conspecific male mouse was introduced into the test subject’s home cage for a 5-min period. The social interaction between the subject and the juvenile intruder was video-recorded and the length of time spent engaged in social investigation was determined from the video records. Social behavior was determined as the amount of time that the experimental subject spent investigating (e.g., anogenital sniffing and trailing) the intruder (Fishkin and Winslow, 1997). The results were expressed as the ratio between the time devoted to social behavior after the treatment, and the respective baseline response.

### Mouse Blood and Brain Tissue Samples

After completion of the behavioral test, 6- and 12-month-old SAMP8 and SAMR1 mice were decapitated to collect trunk blood. Blood was allowed to clot at 4°C for 30 min and centrifuged to obtain serum. The cerebral cortex and hippocampus were immediately dissected on a cold plate. Samples of blood serum and cerebral tissues were stored at −80°C for further analysis. All samples were obtained 3 h after LPS or vehicle injection. Brain tissue of 9-month-old 5XFAD (*n* = 4) and WT mice (*n* = 4) was also obtained and stored at −80°C until analysis.

### Human Tissue Samples

Brain tissue samples of sEOAD, ADAD and neurologically healthy controls (NHC) were obtained from the Neurological Tissue Biobank of Hospital Clínic – IDIBAPS and the Neuropathology Institute of the Hospital Universitari de Bellvitge. All subjects or their legal representatives provided written consent for the use of the brain samples and the study was approved by the Ethics Committee of the Hospital Clínic of Barcelona. All procedures were conducted in accordance with the 1964 Declaration of Helsinki and its later amendments. Postmortem brain tissue was evaluated following standardized pathological procedures and each patient’s disease was classified according to international consensus criteria (Hyman et al., 2012; Montine et al., 2012). Following these criteria, AD patients included in the study were classified as A3, B3, and C3. All patients were previously screened for the APOE genotype and for mutations in the PSEN1, PSEN2, and APP genes (Antonell et al., 2013). Eight samples per group were used for this study. Characteristics of the subjects are displayed in Table 1. ADAD subjects bore PSEN1 mutations (L286P, V89L, M139T and E120G). The three groups were balanced for sex, postmortem delay and APOE genotype. ADAD and NHC groups were also matched by age, but sEOAD was significantly older than the other two groups [one-way ANOVA, *F*(2,21) = 12.92, *p* < 0.001; sEOAD vs. ADAD *p* < 0.01 and sEOAD vs. NHC, *p* < 0.001].

**TABLE 1:** Characteristics of the human subjects included in the study.

Subject	Sex	Age (y)	*APOE* genotype	PMD (h:min)	FAD mutation	Group
E1	Male	57	4/3	4:30	−	sEOAD
E2	Male	61	3/3	19:15	−	sEOAD
E3	Female	63	3/3	9:00	−	sEOAD
E4	Male	60	3/3	5:00	−	sEOAD
E5	Female	74	3/3	9:00	−	sEOAD
E6	Male	69	3/3	3:30	−	sEOAD
E7	Male	68	3/3	9:00	−	sEOAD
E8	Female	65	3/3	16:00	−	sEOAD
		65 ± 1.8		9:24 ± 1:50		
P1	Female	56	3/3	5:00	*PSEN1 (L286P)*	ADAD
P2	Male	54	3/3	7:30	*PSEN1 (V89L)*	ADAD
P3	Male	64	3/3	14:45	*PSEN1 (M139T)*	ADAD
P4	Male	57	3/2	9:30	*PSEN1 (V89L)*	ADAD
P5	Male	44	3/3	5:30	*PSEN1 (E120G)*	ADAD
P6	Male	53	3/3	5:15	*PSEN1 (M139T)*	ADAD
P7	Female	48	4/3	16:25	*PSEN1 (M139T)*	ADAD
P8	Male	57	3/3	15:15	*PSEN1 (M139T)*	ADAD
		54 ± 2.0		9:54 ± 1:36		
C1	Female	45	3/3	14:40	−	NHC
C2	Male	46	3/2	9:35	−	NHC
C3	Male	47	3/3	4:55	−	NHC
C4	Male	49	3/3	7:35	−	NHC
C5	Male	53	3/3	7:25	−	NHC
C6	Male	58	4/3	4:00	−	NHC
C7	Male	59	3/3	6:25	−	NHC
C8	Male	50	3/3	12:00	−	NHC
		51 ± 1.8		8:18 ± 1:12		

**For each group, mean ± SEM of age and PMD are shown. ADAD, autosomal dominant Alzheimer’s disease; FAD, familial Alzheimer’s disease; NHC, neurological healthy control; PMD, postmortem delay; sEOAD, sporadic early-onset Alzheimer’s disease*.*

### RNA Extraction and qPCR Analysis

Total RNA was isolated from mouse tissue samples using the mirVana^TM^ miRNA Isolation Kit with phenol (Applied Biosystems, Foster City, CA, United States) in accordance with the manufacturer’s instructions for obtaining total RNA, including small RNA. Isolation of total RNA from frozen brain tissue of the posterior cingulate area at the thalamus level was performed using an RNeasy Lipid Tissue Mini Kit (Qiagen, Hilden, Germany). RNA yield, purity, and quality were determined using a NanoDrop^TM^ ND1000 spectrophotometer (Thermo Fisher Scientific, Waltham, MA, United States). RNAs with a 260/280 ratio of >1.9 were selected. Random-primed cDNA synthesis was performed using the High-Capacity cDNA Archive kit (Applied Biosystems). Gene expression was measured in a CFX96 Real-Time qPCR Detection System (Bio-Rad, Hercules, CA, United States), using specific TaqMan FAM-labeled probes (Applied Biosystems). The expression of the genes *Il1b*, *Il6* and *Tnf*, coding for the common pro-inflammatory cytokines IL1β, Interleukin 6 (IL6), and tumor necrosis factor α (TNFα), respectively, were analyzed in SAMP8 and SAMR1 cortex and hippocampus. The expression of the mouse genes *Chil1* and *Trem2* that code for CHI3L1 and TREM2, respectively, were analyzed in the hippocampus of SAMP8, SAMR1, 5XFAD, and WT mice. The human counterpart genes *CHI3L1* and *TREM2* were analyzed in human samples of the posterior cingulate area at the thalamus level. Mouse data were normalized to *TBP* gene expression and human data to *PGK1* and *B2M*. mRNA levels were expressed as fold change of the control group (6-month-old SAMR1 treated with vehicle, WT mice or NHC, as appropriate). A list of primers utilized is presented in Table 2.

**TABLE 2:** Genes analyzed and TaqMan FAM-labeled probes for real-time qPCR.

Function	Symbol	Full name	TaqMan Assay	NCBI RefSeq	Species
Pro-inflammatory cytokine	*Il1b*	Interleukin 1β	Mm00434228_m1	NM_008361.3	Mouse
	*Il6*	Interleukin 6	Mm00446191_m1	NM_031168.1	Mouse
	*Tnf*	Tumor necrosis factor α	Mm00443258_m1	NM_001278601.1 NM_013693.3	Mouse
Unknown, astrogliosis	*Chil1*	Chitinase like protein 1 or YKL-40	Mm00801477_m1	NM_007695.3	Mouse
	*CHI3L1*	Chitinase 3 like protein 1 or YKL-40	Hs01072228_m1	NM_001276.2	Human
Immune response, microgliosis	*Trem2*	Triggering receptor expressed on myeloid cells 2	Mm00451744_m1	NM_031254.3	Mouse
	*TREM2*	Triggering receptor expressed on myeloid cells 2	Hs00219132_m1	NM_001271821.1 NM_018965.3	Human
Reference gene	*Tbp*	TATA-box binding protein	Mm00446971_m1	NM_013684.3	Mouse
	*B2M*	β-2-Microglobulin	Hs00187842_m1	NM_004048.2 XM_005254549.3	Human
	*PGK1*	Phosphoglycerate kinase 1	Hs00943178_g1	NM_000291.3	Human

### Morphology of Cultured Glial Cells

The activated phenotype of glial cells was confirmed by microscopic examination of stained cultures. Glial cells grown on glass coverslips were fixed with 4% paraformaldehyde and astrocytes and microglia were specifically stained using standard histological procedures. Astrocytes were immunostained with the primary antibody to Glial fibrillary acidic protein (GFAP) (1:500; Dako Z0334; Agilent, Santa Clara, CA, United States) followed by the fluorescent Alexa Fluor 546 species-specific conjugated secondary antibody (1:1000; Thermo Fisher Scientific, Waltham, MA, United States). Microglia were stained with lectin from *Bandeiraea simplicifolia* conjugated with fluorescein (1:400; Sigma, L2895). Coverslips were mounted upside down on glass slides with Mowiol 4-88 (Sigma 81381) media. Astrocyte cultures from SAMR1 and SAMP8 showed similar basal morphology and similar morphological transformation in response to LPS + IFN. Microglia proliferation and relative number of cells with morphological changes were analyzed by cell count in microphotographs using Cell F software (Olympus, Shinjuku, Tokyo, Japan).

### Nitrite Assay

Nitric oxide generation by activated glia in culture was measured by the colorimetric Griess reaction that detects nitrite (NO_2_^–^), a stable reaction product of nitric oxide and molecular oxygen. Briefly, 100 μL of conditioned medium were incubated with 100 μL of Griess reagent for 10 min at room temperature. The optical density was measured at 540 nm using a microplate reader (iEMS Reader MF; Labsystems, Vantaa, Finland). The nitrite μM concentration was determined from a sodium nitrite standard curve.

### Cytokine Determinations

The levels of the pro-inflammatory cytokines IL1β, IL6, and TNFα were determined in homogenates of SAMP8 and SAMR1 cerebral cortical tissue and in conditioned culture media. The protein concentration of tissue homogenates was determined using the Bradford assay. IL1β in the serum of the mice was also determined to identify the peripheral response to LPS. Cytokines were determined using commercial ELISA kits, following the manufacturer’s instructions. Mouse IL1β Quantikine ELISA Kit (MLB00C) was purchased from R&D Systems (Minneapolis, MI, United States), Murine IL6 ELISA Set (861.020.005) from Diaclone (Besançon, France) and Mouse TNFα ELISA Ready-SET-Go (88-7324-22) from Thermo Fisher. Samples were measured at 450 nm using a plate reader (iEMS Reader MF; Labsystems, Vantaa, Finland). Data were expressed as pg/ml of blood serum or pg/mg of protein, as appropriate.

### Statistical Analysis

The results are expressed as mean ± SEM. The distribution of the data was checked with the Shapiro–Wilk test, and data were log-transformed into normality where required. Data from the SAMP8 and SAMR1 mice with two factors (strain and treatment in the *in vitro* experiments) or three factors (strain, treatment and age in the *in vivo* experiments) were analyzed by ANOVA to obtain the respective main effects and interaction effects. All ANOVA factors have two levels and therefore no further analysis was performed in the absence of statistically significant interaction. Experimental groups were compared using Fisher’s least significant difference (LSD) test when there was an interaction between factors. Student’s *t*-test was used for comparison between 5XFAD and WT mice. The human tissue results were analyzed by one-way ANOVA followed by LSD *post hoc* test. Statistical analyses were performed using IBM SPSS Statistics v22.

## Results

### Inflammatory Phenotype of SAMP8 Mice

**FIGURE 1:**
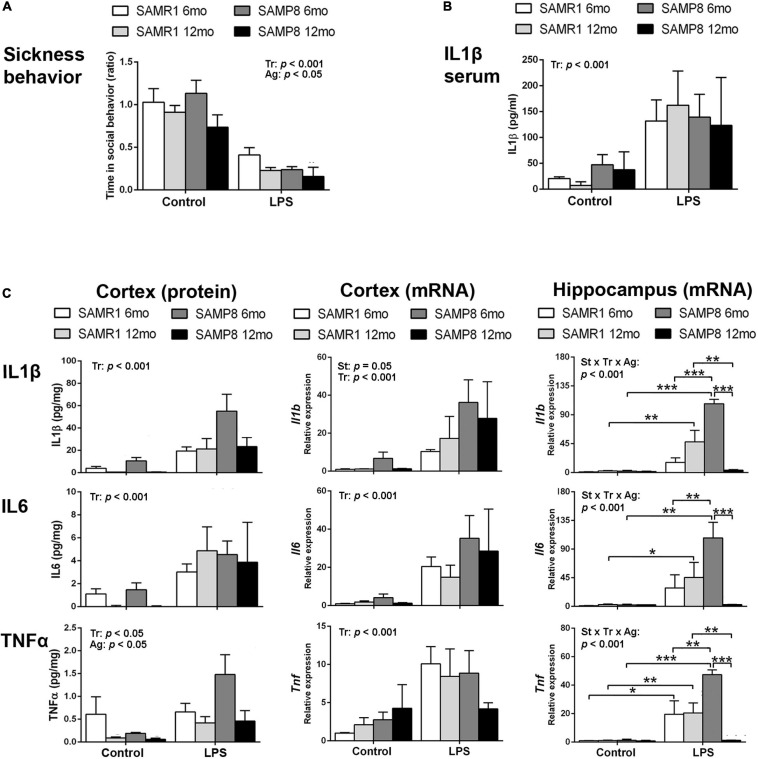
Hippocampus of SAMP8 mice revealed an exacerbated acute inflammatory response to lipopolysaccharide (LPS) at young age and null responsiveness at old age. Sickness behavior 3 h after 3 mg/kg i.p. of LPS showed the effect of treatment and a lowering effect of age in the response of both SAMR1 and SAMP8 mice **(A)**. Blood serum levels of Interleukin 1β (IL1β) were increased in all groups of mice treated with LPS for 3 h regardless of strain and age **(B)**. Cerebral cortical levels of protein and mRNA of pro-inflammatory cytokines Interleukin 1β (IL1β/Il1b, protein/gene), Interleukin 6 (IL6/Il6, protein/gene) and Tumor necrosis factor α (TNFα/Tnf, protein/gene) were generally elevated by LPS in all mice, with lower protein levels of TNFα in all aged mice and higher mRNA levels of Il1b in SAMP8 mice; mRNA levels of all cytokines in the hippocampus showed exacerbated levels in 6-month-old SAMP8 compared to SAMR1 mice and low levels of cytokines indicative of null responsiveness to a pro-inflammatory injury in 12 month-old SAMP8 mice 3 h after LPS injection **(C)**. *P*-values for two-way ANOVA analysis are indicated at the top area of the graph: St, strain main effect; Tr, treatment main effect; Ag, age main effect; and St × Tr × Ag, interaction effect. *P*-values for Fisher’s LSD *post hoc* tests are indicated as: *p < 0.05; **p < 0.01, ***p < 0.001 compared to the corresponding SAMR1 group, the corresponding control treatment group, or the corresponding younger mice group, as indicated. *N* = 3–10 mice/group.

Lipopolysaccharide induced a reduction in social exploration indicative of sickness behavior as expected, showing the effectiveness of the treatment in 6- and 12-month-old SAMR1 and SAMP8 mice. The results 3 h after the injection are depicted in Figure 1A. SAMR1 and SAMP8 mice showed similar responses to LPS injection. Furthermore, both SAMR1 and SAMP8 showed lower social exploration with age [three-way ANOVA, main effect of treatment: *F*(1,44) = 55.086, *p* < 0.001; main effect of age: *F*(1,44) = 4.323, *p* = 0.043].

Protein levels of the first-line pro-inflammatory cytokine IL1β in the blood serum of these animals are shown in Figure 1B. There was a significant increase in response to LPS stimulus in all groups, without effect of strain or age [three-way ANOVA, main effect of treatment: *F*(1,18) = 18,371, *p* < 0.001].

Protein and mRNA levels of the pro-inflammatory cytokines IL6, IL1β, and TNFα for these mice 3 h after the injection of LPS or vehicle are shown in Figure 1C. Protein levels of IL1β, IL6, and TNFα generally increased in the cerebral cortex of SAMR1 and SAMP8 mice in response to LPS systemic injury as indicated by statistical significance of treatment in ANOVA [three-way ANOVA, main effect of treatment: *F*(1,51) = 15.639, *p* < 0.001 for IL1β; *F*(1,42) = 15,475, *p* < 0.001 for IL6; and *F*(1,48) = 5.179, *p* = 0.027 for TNFα]. However, the results of 6-month SAMR1 protein levels and 12-month SAMP8 mRNA levels showed no tendency to increase those of TNFα after treatment with LPS. Furthermore, TNFα levels generally decreased with age [three-way ANOVA, main effect of age: *F*(1,48) = 7.165, *p* = 0.010 for TNFα]. A visible trend for lower IL1β and IL6 levels in the control treatment in 12-month-old mice compared to 6-month-old mice for both strains did reach significance when vehicle-injected mice were analyzed separately from LPS groups (not indicated in the figure) [two-way ANOVA, main effect of age: *F*(1,24) = 7.723, *p* = 0.010 for IL1β; *F*(1,22) = 10.257, *p* = 0.004 for IL6].

LPS induced increased mRNA levels of *Il1b*, *Il6*, and *Tnf* in the cerebral cortex of both strains of mice [three-way ANOVA, main effect of treatment: *F*(1,30) = 40.574, *p* < 0.001 for *Il1b*, *F*(1,33) = 28.382, *p* < 0.001 for *Il6*, and *F*(1,30) = 19.163, *p* < 0.001 for *Tnf*]. Furthermore, SAMP8 showed higher levels of *Il1b* mRNA [main effect of strain: *F*(1,30) = 4.159, *p* = 0.050 for *Il1b*].

In the hippocampus, LPS also induced an increase in the mRNA of the three cytokines [three-way ANOVA, main effect of treatment: *F*(1,23) = 27.187, *p* < 0.001 for *Il1b*, *F*(1,22) = 30.821, *p* < 0.001 for *Il6*, *F*(1,23) = 34.536, *p* < 0.001 for *Tnf*]. However, the levels attained were very high in the 6-month-old SAMP8 mice compared to SAMR1 and to the corresponding levels in SAMP8 cerebral cortical tissue. Furthermore, 12-month-old SAMP8 mice showed significantly lower mRNA levels of the three cytokines than in 6-month-old SAMP8 mice after LPS injection [three-way ANOVA, main effect of age: *F*(1,23) = 4.839, *p* = 0.038 for *Il1b* and *F*(1,23) = 10.082, *p* = 0.004 for *Tnf*; effect of treatment × strain × age interaction: *F*(1,23) = 17.257, *p* < 0.001 for *Il1b*, *F*(1,22) = 4.326, *p* = 0.049 for *Il6*, *F*(1,23) = 10.542, *p* = 0.004 for *Tnf*].

Overall, SAMP8 mice were similarly responsive than SAMR1 mice against LPS injection at the peripheral level. However, there was a differential response in the brain tissue. Specifically, SAMP8 mice showed a general increase of *Il1b* expression in the cerebral cortex and, most noticeably, an extreme activation of the gene expression of *Il1b*, *Il6* and *Tnf* induced by LPS in the hippocampus at young age followed by null responsiveness to infection-like stimulus at old age. Therefore, there was an exacerbated immune response in young SAMP8 and immunosuppression in aged SAMP8 hippocampi.

### Inflammatory Phenotype of SAMP8 Glial Cultures

**FIGURE 2:**
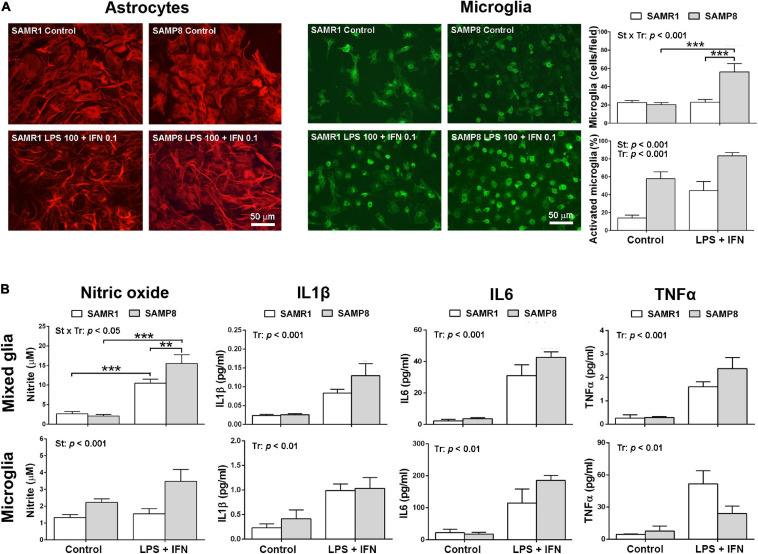
Mixed glial cultures of SAMP8 senescent mice had a pro-inflammatory phenotype mainly driven by microglia. Representative images of astrocytes stained with glial fibrillary acidic protein (GFAP) and microglia cultures stained with lectin in mixed glial cultures in control conditions or submitted to a 24-h treatment with lipopolysaccharide (LPS) 100 ng/ml + interferon γ (IFN) 0.1 ng/ml; histograms of the average cell counts of microglia per microscopic field and the percentage of microglia with reactive phenotype shown by globular morphology, as indicated **(A)**. Histograms of the nitric oxide generation, and the levels of pro-inflammatory cytokines Interleukin 1β (IL1β), Interleukin 6 (IL6) and Tumor necrosis factor α (TNFα) released into the culture media of mixed cultures and microglia cultures, in control conditions or treated with LPS 100 ng/ml + IFN 0.1 ng/ml, as indicated **(B)**. *P*-values for two-way ANOVA analysis are indicated at the top area of the graph: St, strain main effect; Tr, treatment main effect; and St × Tr, interaction effect. *P*-values for Fisher’s LSD *post hoc* tests between the groups indicated in the graph are as follows: **p < 0.01, ***p < 0.001. *N* = 6–10 (microglia micrographs) from 3 independent cultures per group for analysis of microglia number and phenotype in mixed glial cultures, *N* = 18–24 (mixed glia)/15–26 (microglia) from 4 to 6 independent cultures per group for nitrite determination and *N* = 4–8 (mixed glia)/3–6 (microglia) independent cultures per group for cytokine analysis. Scale bar = 50 μm.

Phenotypic images of astrocytes and microglia in mixed glial cultures from SAMR1 and SAMP8 mice are shown in Figure 2A. SAMP8 astrocytes were visualized by immunostaining of the GFAP marker in mixed glial cultures highly enriched in these cells. In control conditions, SAMP8 astrocytes had a similar morphology to SAMR1 astrocytes. Both astrocyte strains acquired a typical activated morphology with filiform processes after pro-inflammatory injury induced by LPS + IFN treatment. However, SAMP8 microglia stained with lectin showed a globular morphology indicative of the activated state in control conditions similar to that of SAMR1 microglia after LPS + IFN injury. Pro-inflammatory treatment also induced proliferation in SAMP8 microglia, but not in SAMR1 microglia, as confirmed by cell count (upper histogram, Figure 2A) [two-way ANOVA, main effect of strain: *F*(1,29) = 11.42, *p* = 0.0021, main effect of treatment: *F*(1,29) = 15.26, *p* = 0.0005, and effect of strain × treatment: *F*(1,29) = 14.72, *p* = 0.0006]. Furthermore, the morphological transformation appeared more intense in SAMP8 microglia treated with LPS + IFN than in the SAMR1 counterparts. Cell counts revealed a higher percentage of activated microglia in SAMP8 under both control and pro-inflammatory conditions (lower histogram, Figure 2A) [two-way ANOVA, main effect of strain: *F*(1,29) = 39.19, *p* < 0.0001, main effect of treatment: (1, 29) = 17.97, *p* = 0.0002].

Pro-inflammatory physiological changes in SAMR1 and SAMP8 cultures were evaluated by the levels of nitric oxide and cytokine release. The results are shown in Figure 2B. Mixed glial cultures increased nitric oxide generation following treatment with LPS + IFN, as determined by nitrite accumulation in the culture media. This response was higher in SAMP8 cultures than SAMR1 cultures [two-way ANOVA, main effect of treatment: *F*(1,74) = 89.93, *p* < 0.0001; main effect of strain: *F*(1,74) = 3.992, *p* = 0.0494; effect of strain × treatment interaction: *F*(1,74) = 6.255, *p* = 0.0146]. SAMP8 pure microglia cultures also showed higher nitric oxide generation than SAMR1 microglia, although the effect of treatment or strain × treatment interaction did not reach significance [two-way ANOVA, main effect of strain: *F*(1,75) = 12.61, *p* = 0.0007]. However, there was a tendency for a difference between nitric oxide levels after LPS + IFN injury in SAMR1 and SAMP8 microglia that was significant by a direct Student’s *t*–test comparison (not indicated in the figure) [*t*(44) = 2.706, *p* = 0.010].

As a whole, glial cell cultures obtained from newborn brain of SAMP8 mice showed higher responsiveness to a pro-inflammatory LPS + IFN injury than those of SAMR1 mice. This effect was primarily driven by microglia, as demonstrated by nitric oxide generation in control and stimulated conditions in pure microglia cultures. Furthermore, the morphological analysis confirmed a state of basal activation, as shown by a higher number of cells with globular morphology, and a hyperreactivity to LPS + IFN, as shown by an increase in the total number and in the percentage of activated SAMP8 microglia.

### Alzheimer’s Disease Neuroinflammatory Markers

**FIGURE 3:**
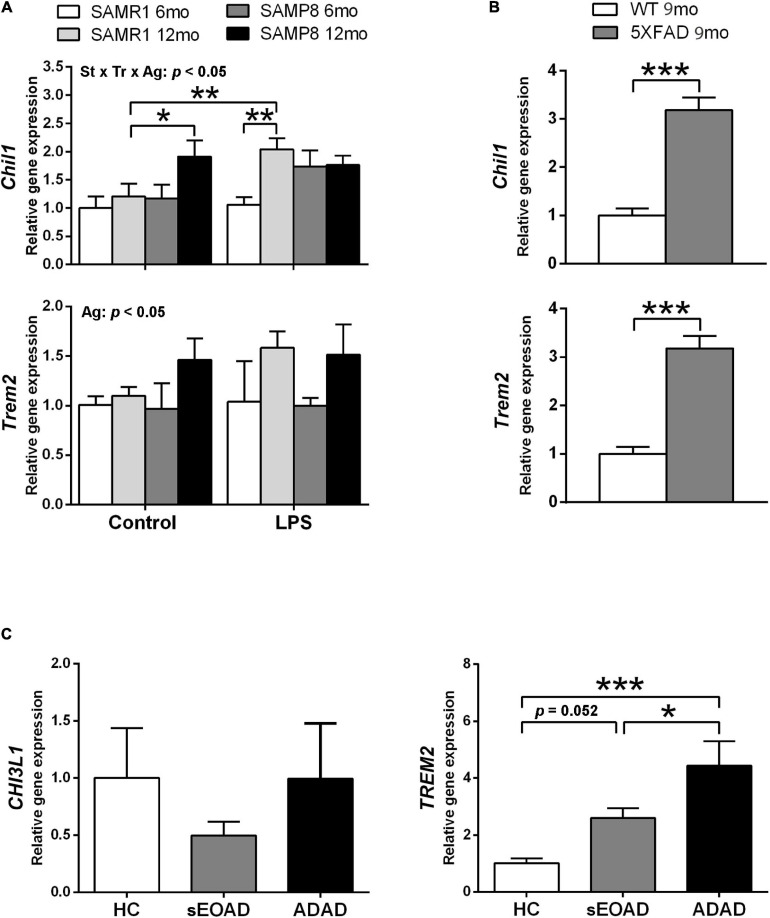
Gene expression of Alzheimer’s disease (AD)-associated markers YKL40, also known as Chitinase 3-Like 1 (CHI3L1/Chil1, human/mouse gene), and Triggering receptor expressed on myeloid cells (TREM2/Trem2, human/mouse gene) showed differential patterns in the hippocampus of SAMP8 mice in comparison with AD samples. Trem2 expression showed a general increase with age in SAMP8 and SAMR1 mice, whereas Chil1 showed a greater increase with age in SAMP8 under control treatment and in SAMR1 3 h after 3 mg/kg i.p. of lipopolysaccharide (LPS) **(A)**. Both Chil1 and Trem2 genes were highly expressed in the hippocampus of the AD mouse model 5XFAD **(B)**. The respective human homologous gene CHI3L1 showed no changes of expression in the posterior cingulate cortex of early onset AD (sEOAD) or autosomal dominant AD (ADAD) as compared to neurologically healthy controls (NHC), whereas TREM2 showed increased expression in AD **(C)**. *P*-values for three-way ANOVA analysis are indicated at the top area of the graph (Ag, age main effect; and St × Tr × Ag, strain × treatment × age interaction effect) and *p*-values for Fisher’s LSD *post hoc* tests between the groups indicated in the graph are indicated as follows: *p < 0.05 and **p < 0.01, *N* = 3–7, in **(A)**. *P*-values for *t*-test are indicated as: ***p < 0.001, *N* = 4, in **(B)**. *P*-values for LSD tests after one-way ANOVA are indicated as: *p < 0.05 and ***p < 0.001, *N* = 8, in **(C)**.

Phenotypic gene expression of *Chil1* and *Trem2* in SAMR1 and SAMP8 hippocampus 3 h after acute LPS injury or control conditions is shown in Figure 3A. Both *Chil1* and *Trem2* mRNA increased with age, although the effect was almost null in the vehicle-injected SAMR1 mice [three-way ANOVA, main effect of age: *F*(1,25) = 7.602, *p* = 0. 011 for *Chil1*, and *F*(1,24) = 7.174, *p* = 0.013 for *Trem2*]. Meanwhile, LPS treatment induced *Chil1* expression in 12-month-old SAMR1 mice but not in the younger counterparts, whereas LPS-treated SAMP8 mice at both ages showed similar expression than control 12-month-old SAMP8 [three-way ANOVA, effect of treatment × strain × age interaction: *F*(1,25) = 4.386, *p* = 0. 047 for *Chil1*]. The variability of the *Trem2* data did not allow obtaining further ANOVA significant results in addition to the main effect of age; however, there was a tendency for a difference between 12-month-old SAMR1 in control conditions and LPS treatment that was significant by a direct Student’s *t*–test comparison (not indicated in the figure) [*t*(11) = 2.669, *p* = 0.0218].

The AD mouse model 5XFAD showed greater hippocampus expression of both *Chil1* and *Trem2* than WT mouse siblings, as shown in Figure 3B [*t*(6) = 7.39, *p* < 0.001, for *Chil1* and *t*(6) = 7.39, *p* < 0.001 for *Trem2*].

The mRNA levels of the corresponding human genes *CHI3L1* and *TREM2* in posterior cingulate tissue samples are shown in Figure 3C. No significant differences were obtained between the NHC and AD groups for *CHI3L1*. However, TREM2 mRNA levels were higher in the AD groups than the NHC group. This increase was of borderline significance for sEOAD but highly significant for ADAD. Furthermore, ADAD showed higher *TREM2* expression than sEOAD [one-way ANOVA, *F*(2,21) = 9.839, *p* = 0.001].

Here we found a differential expression in the respective markers for astrogliosis (*Chil1*/*CHI3L1*) and microgliosis (*Trem2*/*TREM2*) in the several brain tissues analyzed from mouse models and AD patients. SAMP8 hippocampus showed increased expression of *Chil1* at old age, although the increase of *Trem2* did not reach statistical significance compared to SAMR1. sEOAD patients showed also a borderline significance in the increase of *TREM2*. However, ADAD patients and 5XFAD mice showed a distinct increased expression of *Trem2*/*TREM2*. 5XFAD mice also showed high levels of *Chil1* expression.

## Discussion

### SAMP8 Mice Showed Dysregulation of the Neuroimmune Response to an Acute Stimulus in the Hippocampus

The SAMP8 mouse model of accelerated brain aging and sporadic AD traits showed a higher neuroinflammatory profile compared to SAMR1, as expected from previous reports (Tha et al., 2000; Álvarez-López et al., 2014; Griñán-Ferré et al., 2016a; Rancán et al., 2017). However, we found a major dysregulation of the neuroimmune response to an acute stimulus in the SAMP8 hippocampus that ranged from hyperresponsiveness at young age to null responsiveness at older age. It is known that the main pro-inflammatory cytokines IL1β, IL6, and TNFα are transiently overexpressed in various regions of mouse brain after an LPS injury (Layé et al., 1994; Catorce and Gevorkian, 2016). Accordingly, both SAMP8 and SAMR1 mice were responsive to LPS by showing an increase in the protein and mRNA levels of these cytokines in the cerebral cortex. A similar response to LPS was also found at the peripheral level. Interestingly, the hippocampus analysis showed a much higher inflammatory response in 6-month-old SAMP8 mice than in the SAMR1 mice of the same age. Furthermore, 12-month-old SAMP8 hippocampus showed no responsiveness to LPS challenge, whereas SAMR1 mice conserved a response of increased cytokine gene expression. Two previous studies performed in young mice reported either no strain differences after a lower dose of 20 μg/kg body weight LPS (Tha et al., 2000) or higher protein levels of the NLRP3 inflammasome in whole brain tissue and IL6 in blood serum of SAMP8 than SAMR1 after 0.33 mg/kg body weight LPS (Ito et al., 2020). Therefore, both young age and a strong LPS challenge may be required to cause an exacerbated generation of pro-inflammatory mediators in SAMP8. Likewise, an insufficient resolution response to age-related inflammation has been reported in SAMP8 hippocampus (Wang et al., 2015b). In fact the hippocampus is highly vulnerable to deterioration by neurotoxic activation of glial cells (Ojo et al., 2015). Accordingly, genome-wide association studies have shown that immune response and related pathways are highly activated in AD brain, suggesting that an increasingly deregulated response with age and infections may result in progressive neurodegeneration and AD (Liu and Yu, 2019). We speculate that chronic neuroinflammation in the SAMP8 hippocampus causes a deregulation of the local neuroimmune response as shown by hyperreactivity to an infectious-like stimulus in young adulthood age and immunosuppression in old age; these processes would contribute to memory loss and neurodegeneration.

### SAMP8 Microglia From Neonatal Mice Showed a Pro-inflammatory State *in vitro*

The *in vitro* analysis showed a higher contribution of microglia than astrocytes in the SAMP8 neuroinflammatory processes. SAMP8 microglia generally showed the amoeboid morphology that is associated with an activated state (Aldana, 2019). However, SAMP8 astrocytes did not show overt reactive phenotype in control conditions, although they have shown some functional deficiencies *in vitro* (García-Matas et al., 2008, 2015). Experiments in cell cultures have shown that both microglia and astrocytes are able to synthesize IL1β, IL6 and TNFα (Lam et al., 2017; Rodgers et al., 2020). Here there was a similar response to LPS + IFN injury in the release of pro-inflammatory cytokines in mixed glial cultures enriched in astrocytes and in microglia cultures. However, we found higher nitric oxide production in LPS-challenged SAMP8 mixed glia and more crucially, in SAMP8 pure microglia regardless of treatment conditions. These effects were paralleled by increased proliferation and morphological changes of SAMP8 microglia. Excessive nitric oxide generated by microglia through sustained expression of inducible nitric oxide synthase (iNOS) is believed to contribute to age-associated neurodegeneration (Yuste et al., 2015). Accordingly, higher nitric oxide content (Wang et al., 2013) and iNOS expression (Griñán-Ferré et al., 2016a) have been reported in SAMP8 brain tissue than in SAMR1. Neuroinflammatory priming has also been reported in microglia isolated from the brains of adult SAMP8 as compared to SAMR1 mice (Ito et al., 2020). Exacerbated inflammatory responses in neurodegenerative disorders are mainly attributed to microglia, in a proposed vicious cycle of sustained microglial activation, neuroinflammation and neurodegeneration (Aldana, 2019; Ahmad et al., 2019). Nevertheless, there is a current consent on the complex dynamics and heterogeneity of microglia activation beyond the initially proposed pro-inflammatory M1 (classical activation) and immunosuppressive M2 (alternative activation) states (Ransohoff, 2016; Song and Suk, 2017; García-Revilla et al., 2019). Microglia polarization can also evolve to alternative activated phenotypes involved in repair functions (Colton et al., 2006; Masuda et al., 2019). Specifically, a subset of microglia, the “disease-associated microglia” (DAM), may be able to sense neuronal damage signals from AD and other conditions and promote phagocytosis, barrier formation and activation of protective pathways (Deczkowska et al., 2018). Furthermore, the presence of microglia with a senescent phenotype in the hippocampus of 12-month-old SAMP8 would be consistent with the senescent dystrophic microglia that have been described in the human aging brain (Streit et al., 2004). Senescent microglia no longer perform their functions of immune surveillance and response or other active neuronal supportive functions including activity-dependent remodeling of synaptic connections (Fields et al., 2014).

### Gene Expression of the Astrogliosis Marker CHI3L1 Was Increased in SAMP8 and 5XFAD Mice but Not in Alzheimer’s Disease Brains

In the brain, *CHI3L1* is expressed mainly by astrocytes and it increases in regions of neuroinflammation in AD and tauopathies (Querol-Vilaseca et al., 2017), and also in other neuroinflammatory conditions (Bonneh-Barkay et al., 2010). We found increased expression of *Chil1* gene in the hippocampus of 12-month-old SAMP8 and 9 month-old 5XFAD mice, in agreement with the progression of age-related astrogliosis (Girard et al., 2014; Corpas et al., 2017) and the advanced stage of neurodegeneration in both mouse models. Noticeably, *Chil1* expression increased in 12-month-old hippocampus of control SAMR1 mice after LPS challenge. Therefore, the level of neuroinflammation needed to upregulate *Chil1* may require the interaction of advancing age and systemic infection in these mice. Also, increased expression of *Chil1* has been shown in other AD transgenic mouse models at advancing age (Colton et al., 2006; Xiao et al., 2016). In human brain, increased expression of the *CHI3L1* gene has been reported in postmortem tissue samples of sporadic AD patients in their 70s and 80s (Colton et al., 2006; Llorens et al., 2017) and further potentiated by systemic infection (Rakic et al., 2018). However, we found no significant changes in expression in the mRNA of the younger AD cohorts tested despite its severe pathology. Intriguingly, it may be required an older age and/or the presence of infection for detecting increased expression of *CHI3L1* despite the increased protein levels in the CSF of AD patients (Schindler et al., 2019; Antonell et al., 2020).

The function of CHI3L1 protein remains unknown. *In vitro*, soluble inflammatory mediators released by macrophages or microglia have been reported to induce *CHI3L1* transcription and morphological changes in astrocytes (Bonneh-Barkay et al., 2012). The results of an *in vivo* study of traumatic brain injury have suggested that CHI3L1 is protective (Wiley et al., 2015), whereas the results from a study with chronic brain infusion of amyloid beta suggested otherwise (Choi et al., 2018). It is possible that expression of *CHI3L1* with advancing neurodegeneration is deleterious, as shown for instance by its inverse correlation with survival in amyotrophic lateral sclerosis (Sanfilippo et al., 2019).

### Gene Expression of the Microgliosis Marker TREM2 Was Highly Increased in Mice and Alzheimer’s Brain With Familial Disease Type

Upregulation of the *Trem2* gene in SAMP8 hippocampus was detected in aged mice, consistent with a previously reported age-related increase in whole brain protein levels in these mice (Jiang et al., 2014b). Remarkably, the gene upregulation was driven by aging and was statistically unrelated to peripheral challenge with LPS in both SAMP8 and SAMR1. Furthermore, middle-aged 5XFAD mice with advanced AD-like pathology showed robust upregulation of *Trem2* in agreement with previous results reported in this and other transgenic AD mouse models (Jiang et al., 2014a; Brendel et al., 2017; Hüttenrauch et al., 2018). Our results in human AD brain showed increased *TREM2* mRNA in the posterior cingulate brain area of AD patients, although the increase of the sEOAD group did not reach significance compared to NHC. We cannot rule out a contribution of age factor on the small increase of *TREM2* mRNA levels in sEOAD patients, who were slightly older than NHC and ADAD groups. However, previous authors have also shown a trend to increase the overall *TREM2* expression levels in sporadic AD cases in comparison to controls (Roussos et al., 2015; Del-Aguila et al., 2019). Remarkably, we found a differential increase between sEOAD and ADAD patients, namely, gene-driven AD increased *TREM2* expression levels by almost twice as much as sporadic AD, a similar fold difference as in the transgenic 5XFAD mouse model compared to aged SAMP8 mice. Therefore, the increased expression of *TREM2* in ADAD brain could indicate a differential microglia response depending on the AD etiology. What is more, the differential level of expression of *TREM2* does not seem driven by the degree of pathology, since sEOAD and ADAD had a similar high level of neuropathological changes. Gene expression of *TREM2* has not been previously analyzed in ADAD brain, to our knowledge. However, higher gene expression of *TREM2* in the superior temporal gyrus has been reported in AD carriers of a missense mutation of this gene (Roussos et al., 2015). Also, the analysis of the differential expression of gene transcripts for the three TREM2 isoforms (Zhong and Chen, 2019) by RNA-Seq has shown an association between AD cases and higher levels of the shortest transcript that lacks the transmembrane domain, in the parietal lobe of sporadic AD brain and *TREM2* mutation carriers (Del-Aguila et al., 2019). Unfortunately, our qPCR analysis did not discern the different *TREM2* transcripts.

The transmembrane glycoprotein TREM2 is a crucial component of a receptor-signaling complex that regulates the immune response and phagocytic activity of microglia, macrophages, dendritic cells and osteoclasts (Sudom et al., 2018). Furthermore, it is involved in neuroprotection mediated by the DAM phenotype (Deczkowska et al., 2018). *TREM2* gene variants with loss-of-function effects are a known risk factor for late-onset AD (Sudom et al., 2018). However, higher levels of TREM2 protein have been reported in the middle temporal cortices of late-onset AD patients in their 80s than in age-matched control tissue, and this increase was correlated with that of phosphorylated tau and apoptotic markers (Lue et al., 2015). This is consistent with the proposed deleterious effect of TREM2 at advanced stages of AD when it would aggravate neuroinflammation (Karanfilian et al., 2020). Animal studies have shown that brain levels of soluble TREM2 increase during aging in parallel with amyloidosis and microglia activation (Brendel et al., 2017). At middle age, *Trem2* studies of gene modulation showed a protective effect in transgenic AD mice (Jiang et al., 2014a) and SAMP8 mice (Jiang et al., 2014b). TREM2 is emerging as a critical factor in the regulation of complex microglia activation states (García-Revilla et al., 2019).

### Conclusion

Pathological changes of microglia activation in the postmortem AD brain have been described in the pioneering studies of the McGeer’s group in the late 1980s and 1990s (McGeer et al., 1987). Later studies overwhelmingly suggest that reactive microglia play an important role in the neuroinflammatory processes of AD. Age-related microglia changes showing a moderately activated phenotype were subsequently described in the normal human brain (Sheng et al., 1998). There is also current agreement on the AD risk induced by low-grade chronic neuroinflammation in the aging brain.

Here we confirmed the presence of increased expression of *TREM2* in AD microglia and unveiled a differential expression between sporadic and gene-driven AD, despite a similarly severe pathology in human (sEOAD and ADAD) and similarly severe cognitive loss and neuroinflammation in mouse models (SAMP8 and 5XFAD). Unexpectedly, we found no changes in the gene expression of the astrocytic protein CHI3L1 (also known as YKL-40) in the human brain of our cohorts of sEOAD or ADAD, while it is known to increase in sporadic AD at older ages. However, the mouse counterpart Chil1 did increase its gene expression. Furthermore, we found that the SAMP8 mice show early microglia-exacerbated reactivity to an infectious-like stimulus *in vitro* and *in vivo*, followed by immunosuppression at old age in the hippocampus. Therefore, SAMP8 mice open a new scenario in the relationship between the progressive impairment of the innate immune system and the expression of AD-related gliosis markers. However, microglia phenotypic changes and derangement of their innate immune function driven by age-related neuroinflammation and AD pathology are complex and warrant further study using diverse experimental systems, including genetic and non-genetic AD models and human samples.

## Data Availability Statement

The original contributions generated for this study are included in the article results, further inquiries can be directed to the corresponding authors.

## Ethics Statement

The studies involving human participants were reviewed and approved by Ethics Committee of the Hospital Clínic of Barcelona, Barcelona, Spain. Written informed consent was not provided because brain samples were obtained from the Neurological Tissue Biobank of Hospital Clínic - IDIBAPS and the Neuropathology Institute of the Hospital Universitari de Bellvitge. Tissue donations follow the legal procedures for their use in research studies. The animal study was reviewed and approved by the Ethics Committee for Animal Experimentation (CEAA) of the University of Barcelona, Spain.

## Author Contributions

RCr, PK, CSo, JM, RS-V, AA, AL, and CSa contributed to the conception and design of the study. AL and CSa jointly supervised the study. PM-M, RCo, EG-L, MC-T, RCr, and CSa did the experimental analysis and data processing. RCo and CSa wrote the manuscript draft. All authors read and approved the final manuscript.

## Conflict of Interest

The authors declare that the research was conducted in the absence of any commercial or financial relationships that could be construed as a potential conflict of interest.
